# Mortality Predictors in Severe COVID-19 Patients from an East European Tertiary Center: A Never-Ending Challenge for a No Happy Ending Pandemic

**DOI:** 10.3390/jcm11010058

**Published:** 2021-12-23

**Authors:** Amalia-Stefana Timpau, Radu-Stefan Miftode, Antoniu Octavian Petris, Irina-Iuliana Costache, Ionela-Larisa Miftode, Florin Manuel Rosu, Dana-Teodora Anton-Paduraru, Daniela Leca, Egidia Gabriela Miftode

**Affiliations:** 1 Department of Infectious Diseases (Internal Medicine II), Faculty of Medicine, University of Medicine and Pharmacy “Gr. T. Popa”, 700115 Iasi, Romania; darieamalia@gmail.com (A.-S.T.); larisa.miftode@yahoo.com (I.-L.M.); lecadaniela@ymail.com (D.L.); emiftode@yahoo.co.uk (E.G.M.); 2 Department of Internal Medicine I (Cardiology), Faculty of Medicine, University of Medicine and Pharmacy “Gr. T. Popa”, 700115 Iasi, Romania; antoniu.petris@yahoo.ro (A.O.P.); ii.costache@yahoo.com (I.-I.C.); 3 Department of Intensive Care Unit, Infectious Diseases Clinical Hospital, 700115 Iasi, Romania; manuelflorin.rosu@gmail.com; 4 Department of Dentoalveolar Surgery and Anesthesiology, Faculty of Dentistry, University of Medicine and Pharmacy “Gr. T. Popa”, 700115 Iasi, Romania; 5 Department of Mother and Child Medicine, Faculty of Medicine, University of Medicine and Pharmacy “Gr. T. Popa”, 700115 Iasi, Romania; antondana66@yahoo.com

**Keywords:** COVID-19, mortality risk factor, severe pneumonia, D-dimer, C-reactive protein

## Abstract

(1) Background: There are limited clinical data in patients from the Eastern European regions hospitalized for a severe form of Coronavirus disease 2019 (COVID-19). This study aims to identify risk factors associated with intra-hospital mortality in patients with COVID-19 severe pneumonia admitted to a tertiary center in Iasi, Romania. (2) Methods: The study is of a unicentric retrospective observational type and includes 150 patients with severe COVID-19 pneumonia divided into two subgroups, survivors and non-survivors. Demographic and clinical parameters, as well as comorbidities, laboratory and imaging investigations upon admission, treatments, and evolution during hospitalization were recorded. First, we sought to identify the risk factors associated with intra-hospital mortality using logistic regression. Secondly, we assessed the correlations between D-Dimer and C-reactive protein and predictors of poor prognosis. (3) Results: The predictors of in-hospital mortality identified in the study are D-dimers >0.5 mg/L (*p* = 0.002), C-reactive protein >5 mg/L (*p* = 0.001), and heart rate above 100 beats per minute (*p* = 0.001). The biomarkers were also significantly correlated the need for mechanical ventilation, admission to intensive care unit, or multiple organ dysfunction syndrome. By area under the curve (AUC) analysis, we noticed that both D-Dimer (AUC 0.741) and C-reactive protein (AUC 0.707) exhibit adequate performance in predicting a poor prognosis in patients with severe viral infection. (4) Conclusions: COVID-19′s outcome is significantly influenced by several laboratory and clinical factors. As mortality induced by severe COVID-19 pneumonia is considerable, the identification of risk factors associated with negative outcome coupled with an early therapeutic approach are of paramount importance, as they may significantly improve the outcome and survival rates.

## 1. Introduction

The SARS-CoV-2 pandemic (Severe Acute Respiratory Syndrome Coronavirus 2), which is currently in full swing, was first detected in December 2019 in Wuhan, China, and resulted in more than 4 million deaths by the end of July 2021 [1,2]. There is a broad spectrum of clinical manifestations, from asymptomatic forms of the disease to severe pneumonias that evolve with systemic impairment, acute respiratory failure, and death [3]. Although most patients develop asymptomatic, mild, or medium forms of COVID-19, followed by a rapid recovery in about two weeks, a prevalence of up to 28% is reported for severe forms of the disease [4]. Serious respiratory symptoms may be due to viral invasion of type II alveolar epithelial cells, triggered local inflammation, and systemic release of inflammatory markers [5]. The mortality rate is increased for patients with severe COVID-19, while for those requiring admission into intensive care unit (ICU) is even higher, ranging from 35% to 50% [6].

Several studies have researched mortality predictors in patients with SARS-CoV-2-induced pneumonia, and identified age, cardiovascular and metabolic comorbidities, and C-reactive protein (CRP) as significant prognosis factors [7,8]. Genetic factors could also play a major role in the progression of severe forms, dominated by acute respiratory distress syndrome. Opposite effects have been noticed concerning the blood groups: while type A increases the risk, type O exhibits a protective role [9]. Data from the Eastern European area are limited and the characteristics of patients are highly variable between regions, which is why this study emphasizes the detailed description of multiple parameters and includes patients exclusively with severe forms of disease who were approached in a single tertiary center in Iasi, Romania [4].

The decision to intubate and mechanically ventilate patients with COVID-19 is marked by controversy and is performed according to the patient’s condition and through clinical judgement, as there are no evidence-based ventilation strategies to date. Large epidemiological studies report a rate of tracheal intubation in hospitalized patients between 2.3% and 33.1% [10,11]. Adequate timing for oro-tracheal intubation and mechanical ventilation is essential for a favorable outcome, but given the non-standardized protocols, there is increasing uncertainty regarding the optimal timing for introducing invasive ventilation. Some studies claim that early intubation could improve survival, while others find no association between the time of intubation and mortality or the length of stay in the ICU, even after assessing patient comorbidities and the severity of the disease [12,13].

The main objective of this study was to identify risk factors associated with intra-hospital mortality in patients with severe forms of COVID-19 pneumonia. Secondly, we investigated the incidence and type of complications during hospitalization and the number of patients who required invasive ventilation.

We present the results of a comprehensive retrospective analysis that included clinical, laboratory, imaging, therapeutic, and evolution parameters for patients with a severe form of COVID-19 pneumonia. Patients were divided into two groups, survivors and non-survivors, with various relevant parameters being recorded and compared between the two groups. Early identification of risk factors associated with increased mortality could allow timely recognition of patients with severe forms of the disease or characterized by a high-risk profile. A dynamic adaptation of therapeutic strategies comprising these factors can be an important step towards improving patient prognosis.

## 2. Materials and Methods

### 2.1. Study Design, Population Characteristics and Laboratory Investigations

Only patients with a COVID-19 infection that was confirmed by real-time reverse transcription-polymerase chain reaction were included in the study. All parameters were recorded retrospectively from a cohort of 150 patients from the North-East region of Romania admitted to the Infectious Diseases Clinical Hospital in Iasi between June and December 2020. We compared 100 consecutively deceased patients due to a severe form of COVID-19 pneumonia to a lot of 50 consecutively enrolled patients also presenting a severe form of COVID-19 who survived and were discharged in a stable condition. We excluded seven patients with incomplete laboratory analyses. Medical records were reviewed by two independent researchers and later converted into a computerized database. The data collected included demographic and clinical parameters, medical history, laboratory and imaging results, treatment, and evolution during hospitalization.

Laboratory parameters included the assessment of complete blood count, biochemical profile (i.e., liver and renal function, blood glucose, lactate dehydrogenase (LDH)), international normalized ratio (INR), D-dimer, interleukin-6 (IL-6), C-reactive protein (CRP), and ferritin. Only laboratory results from the first 48 h after admission were included in the statistical analysis. A computed tomography (CT) scan was performed in every admitted patient for imaging diagnosis. The abnormalities identified on CT-scan included ground-glass opacities and areas of consolidation. Focal pulmonary infiltration is defined by a consolidation which is limited to a certain lung segment or lobe. Diffuse lung involvement is defined by scattered and randomly distributed abnormalities with no respect to lung segmentation, with the presence of ground-glass opacities and/or consolidation areas. Bilateral pulmonary infiltration was used to describe CT abnormalities affecting both lungs in variable proportion. Severe acute complications that occurred during hospitalization, eventually contributing to the mortality rate, were noted as well.

Two groups of patients were formed according to the clinical outcome, namely survivors and non-survivors, with baseline characteristics being compared accordingly. Oxygen titration devices were used to prevent or treat hypoxemia, under standard pulse oximetry or invasive arterial monitoring. Severe oxygen-refractory hypoxemia on high-flow nasal cannula or non-invasive ventilation by continuous positive airway pressure and severe dyspnoea were considered significant criteria for endotracheal intubation. During mechanical ventilation, large tidal volumes were avoided.

Acute Respiratory Distress Syndrome was defined according to the Berlin definition. Severe disease was defined as peripheral oxygen saturation of 93% in room air at sea level, polypnea (≥30 breaths per minute), or the presence of infiltrates affecting more than 50% of lung parenchyma [14]. Fever was defined as axillary temperature higher than 37.5 degrees Celsius. Acute kidney injury was diagnosed using the KDIGO criteria [15]. Acute cardiac injury was defined as elevated troponin levels above the upper limit of the reference range (>0.1 ng/mL). Septic shock was defined in agreement with the Third International Consensus Definitions for Sepsis and Septic Shock [16].

Patients were treated according to a local protocol that included routine thromboprophylaxis depending on the risk of bleeding, but also, in selected cases, antivirals, corticosteroids, or the IL-6 receptor antagonist Tocilizumab. At the time of the study, the antivirals used were Lopinavir and Ritonavir. Tocilizumab was administered as a single dose in 76% of cases. Only patients with unfavorable evolution received a second dose. The decision to administer Tocilizumab was based on oxygen saturation and inflammatory markers’ values. Corticosteroid therapy consisted of Dexamethasone for at least 10 days. Antibiotics have been used for bacterial co-infections and in immunocompromised patients, with the most commonly used being carbapenems, Linezolid, and fluoroquinolones. Optimizing the glycemic profile was a routine practice, while renal replacement therapy was used in 18 cases.

### 2.2. Statistical Analysis

Descriptive data are expressed as numbers and percentages for categorical variables and as medians (with interquartile ranges (IQRs)) and means (with standard deviations (SDs)) for continuous variables according to distribution. The Two sample T-test and Mann–Whitney U test were used to compare differences between the two groups. T-test was preceded by Levene’s test for categorical variables. Multivariate logistic regression analysis was performed to identify risk factors associated with in-hospital death using the forward selection (likelihood ratio) method. Quality assessment of the logistic regression model was performed using the Hosmer-Lemershow test.

The diagnostic performance of the biomarkers in patients admitted to Intensive Care Unit was evaluated by receiver operating characteristic (ROC) analysis, with the subsequent comparison of the areas under the curve (AUC). The cut-off values for D-Dimers and CRP were also drawn from the ROC curve, using various criterion, as appropriate.

The statistical level of significance was set at 0.05. The SPSS version 23.0 (IBM, Armonk, NY, USA) statistical software was used for conducting all analyses.

### 2.3. Ethics

The data were retrospectively extracted from patients’ medical records. A standard informed consent regarding participation in the study and the further use of personal data for research purposes was signed by all patients at admission, as part of the standard personal medical file, conceived and approved by the board of the hospital. In order to comply with the privacy policy, all patient identification data were removed. The study was conducted according to ethical principles contained in the 1975 Declaration of Helsinki (revised in 2013) and was approved by the University of Medicine and Pharmacy “Grigore T. Popa” of Iasi Ethics Committee (approval number 55, date of approval 8 March 2021)

## 3. Results

### 3.1. Baseline Characteristics

A total of 150 patients were included in the study, of whom 92 (61.3%) were men (Table 1). The average age was 66.4 ±13.3 years, including patients aged between 30 and 95 years. Compared to survivors, non-survivors were significantly older (*p* = 0.002), with more than half of them being over the age of 70. Most of the included patients were in the 60–69 years of age group (32%).

The most prevalent comorbidities were hypertension (60.7%) and obesity (42%), followed by chronic heart failure (41.3%) and diabetes (38.7%) (Table 2). The distribution of comorbidities differed between survivors and non-survivors in terms of cardiovascular diseases, such as high blood pressure (44% vs. 69% *p* = 0.003), coronary artery disease (20% vs. 36% *p* = 0.035), chronic heart failure (30% vs. 47% *p* = 0.042), or atrial fibrillation (8% vs. 21% *p* = 0.023). We have also noted significant differences between the two groups in terms of the history of chronic kidney disease, *p* < 0.001. Diabetes mellitus, chronic obstructive respiratory diseases, cerebrovascular or liver diseases did not differ significantly between the two groups (*p* > 0.05). The most common symptoms identified at the time of admission were dyspnoea and fatigue, followed by cough and fever. Less common symptoms were chills, headache, digestive disorders, and skin rash. Fatigue and chills were significantly more common in the non-survivor group compared to the survivor group (*p* = 0.019 and *p* = 0.021, respectively). Patients in the non-survivor group had a higher heart rate at admission (*p* = 0.022), but no significant differences concerning blood pressure levels.

The median time period from the onset of symptoms to presentation to hospital and admission was 4.4 (IQR 3–6) days, with no significant differences between the two groups. The median length of hospitalization was significantly longer in survivors, 15 (IQR 13–19) days, compared to non-survivors, 10 (IQR 6–14) days (*p* < 0.001). The median duration from admission to the ICU to death was six (IQR 3–8) days.

### 3.2. Laboratory and Imagistic Findings

In terms of laboratory results, compared to survivors, non-survivors had significantly higher levels of IL-6 (*p* = 0.005), neutrophil to lymphocyte ratio (*p* = 0.022), white blood cells (*p* = 0.002), ferritin (*p* < 0.01) and D-dimer (*p* < 0.001) (Table 3).

Computed tomography sections revealed ground-glass opacities in 103 (68.7%) patients. Diffuse and bilateral pulmonary infiltrations were significantly more common in deceased patients compared to survivors (*p* < 0.01).

### 3.3. Therapeutic Approach and Complications

A total of 105 (70%) patients required invasive ventilation, and the mortality rate in patients with orotracheal intubation was 95%. In the survivor group, nine (18%) patients required non-invasive ventilation, while five (10%) patients required invasive ventilation (Table 4). All deceased patients required invasive ventilation, with 52% of them previously receiving non-invasive ventilation. From the total number of patients, 136 (90.7%) received antiviral treatment, 139 (92.7%) received antibiotic therapy, 127 (84.7%) received glucocorticoids, while 125 (83.3%) patients required the administration of Tocilizumab.

During hospitalization, the most common complication was CARDS (coronavirus associated acute respiratory distress syndrome), which was diagnosed in 116 cases (77.3%), followed by multiple organ failure in 39 patients (26%), while acute renal failure was identified in 29 patients (19.3%).

Using a logistic regression model, the following parameters were identified as death predictors (Table 5): D-dimer >0.5 mg/L (*p* = 0.002), CRP > 5mg/L (*p* = 0.001), and heart rate above 100 beats per minute (*p* = 0.001).

### 3.4. Role of Biomarkers in the Assessment of COVID-19 Forms

Consequently, we performed comparisons of D-Dimer and CRP with other relevant predictors of poor prognosis, aiming to assess the potential correlations between these parameters and the analyzed biomarkers (Table 6). We noted that both biomarkers presented a significant and positive correlation with multiple organ dysfunction syndrome, the need for invasive mechanical ventilation, and with the admission to ICU, while only CRP was significantly associated with non-invasive mechanical ventilation. No relationship was found between the use of glucocorticoids or Tocilizumab and the serum levels of the analyzed biomarkers. Moreover, the concentrations of CRP and D-Dimer exhibited a direct and significant correlation with each other.

**Figure 1:**
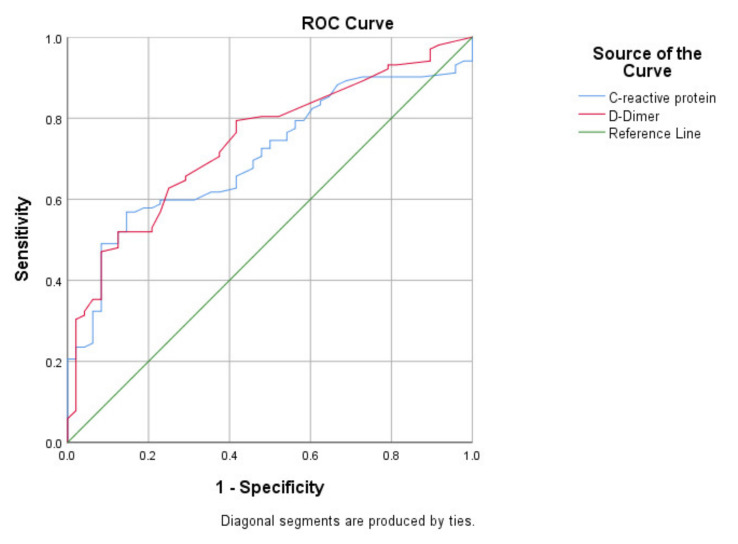
ROC analysis for specified biomarkers.

Based on the significant correlation between the analyzed biomarkers and ICU admission, the next step was to evaluate their performance in predicting a severe course of the disease, by performing a ROC analysis (Table 7). We observed that the curves show an adequate performance for both D-Dimer (AUC: 0.741) and CRP (AUC: 0.707) in predicting admission to ICU (Figure 1).

Further, by using the ROC curve values we aimed to outline several cut-offs for both biomarkers, in order to identify patients at high risk (Table 8).

In our study, a reliable high-risk cut-off for D-Dimer was established at 0.41 mg/L (OR 6.51 (CI 95%: 2.75–15.42), *p* < 0.0001), which is very similar to the international-accepted cut-off of 0.5 mg/L, and characterized by an 80.4% sensitivity and 52.1% specificity. Shifting to more specific (Sp = 92.7%), but less sensitive (Se = 47.1%) value of 2.05 mg/L (Youden’s index), we observed an even increased risk for ICU admission (OR 7.36 (CI 95%: 2.69–20.11), *p* < 0.0001).

Concerning CRP, we identified that a serum concentration of 23 mg/L very well predicts the risk for ICU admission (OR 4.82 (CI 95%: 2.21–10.55), *p* < 0.0001), while a more specific cut-off value of 68.5 mg/L corresponding to Youden’s index (Se = 56.9%, Sp = 85.7%) was basically found almost exclusively among patients admitted in ICU (OR 42.7 (CI 95%: 9.78–185.98), *p* < 0.0001).

## 4. Discussion

The result of this study, which included patients with severe forms of COVID-19 admitted to a tertiary center, is in line with previous reports confirming the independent relationship between CRP, D-Dimer, and mortality in COVID-19 [17,18]. This research adds to a growing body of literature and, to our knowledge, is the first study to include patients from Romania with severe forms of pneumonia caused by SARS-CoV-2.

Epidemiological data reveal that patients over 60 years of age have an increased risk of developing severe forms of COVID-19, with about 80% of deaths occurring among these patients [17]. In our study, we also noticed that patients with fatal outcome were significantly older than their surviving counterparts (*p* = 0.002). Age has been identified as an independent risk factor for mortality in several studies, possibly due to the immune system aging phenomenon and the presence of comorbidities [18]. Two major changes occur in the functioning of the immune system with increasing age. The first is immunosenescence, which affects the ability to control viral load, preventing recognition, immune system activation, and viral clearance. The second is inflammaging, characterized by a chronic increase in systemic inflammation, which leads to an overactive but ineffective immune system [19].

The most common symptom at presentation was fatigue. In contrast to other studies where fever was the main clinical aspect, in this study approximately one half of the patients (50.6%) experienced fever at admission [20]. One explanation could be the advanced age, immunodeficiency (diagnosed in 18% of cases), but also the median time from the onset of symptoms at admission, four days. However, fever was observed during hospitalization in 86% of patients, generally within the first six days. The delay or absence of fever prevents early identification of patients infected with SARS-CoV-2, but an atypical presentation is also common, especially among the geriatric population [21].

The inflammatory syndrome can exacerbate pre-existing cardiovascular diseases and although these have not been identified as predictors of mortality in the study, there were significant differences between groups in terms of their prevalence. Factors that may favour the association between cardiovascular disease and mortality in COVID-19 are endothelial dysfunction, myocardial depression, renin–angiotensin–aldosterone system disorder, or coagulation imbalances [22]. Acute heart failure may occur as the first manifestation of SARS-CoV-2 infection or as a subsequent complication, either by exacerbating pre-existing pathologies or secondary to a newly installed cardiomyopathy [23]. In this study, an acute decompensation was observed in 8% of patients with already diagnosed chronic heart failure in their medical history, a lower figure compared to a 23% prevalence that was reported by other authors [24]. Even if the precise mechanism of heart failure in COVID-19 is yet to be established, a plethora of factors are incriminated. A potential trigger is the imbalance between high cardiac oxygen consumption (due to inflammation-induced tachycardia) and a decreased oxygen supply (hypoxemia secondary to pulmonary injuries). Moreover, the coronary perfusion may be impaired in the context of microvascular ischemia or due to sepsis-associated hypotension, which are common findings among COVID-19 patients. An overlooked risk factor for cardiac decompensation, especially in patients with severe COVID-19 pneumonia, is represented by mechanical ventilation with elevated positive end-expiratory pressures, which induce increased right ventricular strain due to increased afterload [25]. In these patients, routine bedside echocardiography or the assessment of biomarkers able to ascertain right ventricle failure (i.e., ST2) would certainly improve prognosis, as a differential diagnosis with pulmonary embolism should be commonly performed, especially since COVID-19 significantly induces a hypercoagulable state [26].

D-dimers have been identified as important determinants of mortality in our regression model. These fibrin degradation products are the expression of a hypercoagulable status that occurs secondary to the hyperproduction of cytokines and due to direct viral action at the vascular level. The ongoing endothelial dysfunction predisposes to the formation of micro- and macrovascular thrombi, the incidence of pulmonary thromboembolism being estimated at up to 9% [27]. We identified elevated IL-6 levels in all included patients with severe forms, its concentration being significantly different between the two subgroups. Herold et al. reported that maximum serum levels of IL-6 and CRP can predict the need for mechanical ventilation [28], our results confirming this association only for CRP, but not for IL-6. Nevertheless, a surge in the levels of these biomarkers is suggestive for hyper-inflammatory syndrome and may further guide the escalation of treatment and an optimized therapeutic management for critical patients with COVID-19. In this context, increasing interest is shifting towards finding specific, “high-risk” cut-off values for these inflammatory biomarkers. Springer et al. observed that a threshold value of CRP > 40 mg/L performed well in predicting mortality in COVID-19 patients [29], while Liu et al. found that a similar cut-off >41.8 ng/L is associated with a severe course of the disease [30]. Concerning our study, we found that an even lower cut-off value for CRP (23 mg/L) is significantly associated with severe outcome (i.e., ICU admission). D-Dimers represent another well-established predictive factor in COVID-19, with generally accepted high-risk cut-offs ranging from 1 to 3 mg/L [31,32], values which are similar to the results (0.41–2.05) predicting a negative prognosis in patients included in our study.

The neutrophil/lymphocyte ratio is another widely accessible parameter and may be considered a predictor for the critical form of the disease [33]. This ratio differed significantly between the two groups included in our study (*p* = 0.022), but unlike other studies, it was not identified as a predictor of mortality [34]. Neutrophils are known for their contribution to acute pulmonary injuries in viral pneumonias, and their role in COVID-19 could represent an exaggerated version of these pathophysiological mechanisms [35]. The observation is also supported by the fact that the degree of activation of neutrophils is higher in COVID-19 than in pneumonia caused by the influenza virus [36]. Studying neutrophil activation markers shows that they could help identify non-critical patients under an increased risk of developing the critical form of the disease [37]. In line with the previous results from a recent Taiwanese study [38], we observed significant correlations between D-Dimer concentration and the need for mechanical ventilation, both invasive or non-invasive, amongst our patients. Of all the patients included in the study, 105 (70%) required invasive ventilation, 100 (95%) of whom have died. Thus, our results confirm that the mortality rate in patients with severe forms of COVID-19 requiring oro-tracheal intubation was very high, being in line with findings from previous studies [39,40]. Research on identifying the optimal time of intubation in CARDS in order to improve mortality showed different results [12,13]. One study concludes that rapid intubation during hospitalization could improve survival rates in patients with COVID-19 [12]. However, a retrospective cohort found no association between mortality and the time of intubation after admission to the ICU or the use of oxygen therapy through high flow nasal cannula [13]. Graselli et al. showed that low pulmonary compliance (<41 mL/cm H_2_O) together with increased D-dimer increase the mortality rate. The cause could be pulmonary intravascular thrombosis because ventilatory ratio, a dead-space marker, correlates with D-dimer levels [41]. Alternatives to invasive mechanical ventilation are represented by high-flow nasal cannula oxygen therapy (HFNC), continuous positive airway pressure (CPAP), or bilevel positive airway pressure (BiPAP). A recent review turns the spotlight on these ventilation modes, especially in patients with acute respiratory failure (ARF) secondary to COVID-19. Based on extensive literature data, the authors emphasize a step-by-step approach, suggesting that continuous CPAP should be preferred in patients with COVID-19-associated ARF if oxygen therapy via nasal canula is insufficient for adequate oxygenation, and/or if oro-tracheal intubation is not yet indicated, while HFNC therapy is reserved only for patients who cannot tolerate CPAP [42]. This stepwise approach was commonly implemented in our study, the exclusively non-invasive ventilation modes being associated with a better prognosis, irrespective of the ventilation mode. Nevertheless, designing new, standardized guidelines concerning the optimal time for intubation and which ventilation modes could bring a significant benefit are further expected.

The antivirals used at the time of the study, i.e., Lopinavir and Ritonavir, did not have a significant influence on the patients’ evolution. This has also been confirmed by randomized clinical trials, which conclude that treatment does not benefit patients with severe forms of COVID-19 or with already installed acute respiratory distress syndrome [43]. Multiple literature data reveal that corticosteroids can reduce the mortality and duration of mechanical ventilation in patients with CARDS [44,45]. In this regard, several clinical trials determined that a moderate dose of dexamethasone reduced mortality in patients hospitalized with COVID-19 and respiratory failure requiring non-invasive oxygen therapy or even invasive mechanical ventilation. However, the results also indicated that dexamethasone could increase mortality in hospitalized patients who did not require oxygen therapy [45,46,47]. Patients with a severe form of COVID-19 have vastly increased inflammatory markers, as the expression for IL-6 receptor is markedly upregulated in SARS-CoV-2 infection [48,49]. Worth-mentioning, the association of Tocilizumab to Dexametazone reduced mortality by approximately 25% in patients with a severe form of COVID-19 pneumonia [50]. In our research, the use of corticosteroids and Tocilizumab differed significantly between the two groups, *p* = 0.049 and *p* = 0.003 respectively. A retrospective cohort study that included patients admitted in the same university hospital shows efficiency in lowering mortality rates by the use of Tocilizumab in both diabetic and non-diabetic patients [51]. Moreover, the Romanian specialists’ experience in the administration of immunomodulatory treatment early during the onset of cytokine storm demonstrates that it could prevent the progression towards critical forms of the disease [52].

Supportive treatment was fundamental given the current absence of specific and efficient antiviral therapies. Vital functions were monitored and managed using all available resources for respiratory and renal support, but with some inherent limitations, because the center in which patients were treated could not offer cardiac support through extracorporeal membrane oxygenation. Our research identified acute kidney injury (AKI) and acute heart failure as the most common organ complications after acute respiratory. AKI has been reported in up to a quarter of patients in critical condition with SARS-CoV-2 infection, possibly caused by ischemic acute tubular necrosis during systemic collapse. A temporal association has been identified between AKI and acute respiratory failure, both complications being associated with a negative prognosis [53].

### Limitations of the Study

The main limitations were the limited sample size and the unicentric design of the study. Secondly, some patients had a late hospital admission that may have contributed to the unfavorable clinical outcome. Last but not least, the lack of certain mechanical assistance devices for cardiopulmonary support may have influenced the prognosis. Nevertheless, we consider that due to the strict eligibility criteria we applied in our study, the included population is representative of the severe COVID-19 pneumonia cases which were diagnosed and treated in the North-East region of Romania. Knowledge of risk factors associated with mortality is crucial in establishing the prognosis, as identifying predictors at the time of admission helps to select patients and immediately start treatment to prevent the progression to the critical form of the disease.

## 5. Conclusions

D-dimer, CRP, and heart rate have been identified as important mortality predictors in our geographical area. Moreover, specific cut-off values of these biomarkers were associated with a significantly increased risk of ICU admission and a more severe outcome. These results can enhance the development of rapid intervention strategies for patients with severe forms of COVID-19, with a prompt initiation of anti-inflammatory therapies or advanced cardiopulmonary supportive care. Integrative management should be implemented in patients with COVID-19, especially in those with confirmed severe pneumonia, in order to positively impact the mortality rates.

## Figures and Tables

**Table 1:** Baseline characteristics.

Demographics and Clinical Characteristics				
	Total	Survivors	Non-Survivors	
	(*n* = 150)	(*n* = 50)	(*n* = 100)	*p* Value
Age (years)	66.4 (13.3)	61.2 (13.5)	69 (12.5)	0.002
30–39	5 (3.3%)	4 (8%)	1 (1%)	
40–49	17 (11.3%)	10 (20%)	7 (7%)	
50–59	15 (10%)	0	15 (15%)	
60–69	48 (32%)	22 (44%)	26 (26%)	
70–79	38 (25.3%)	12 (24%)	26 (26%)	
>80	27 (18%)	2 (4%)	25 (25%)	
Sex				0.117
Female	58 (38.7%)	15 (30%)	43 (43%)	
Male	92 (61.3%)	35 (70%)	57 (57%)	
Current smoker	46 (30.7%)	18 (36%)	28 (28%)	0.320
Obesity	63 (42%)	16 (32%)	47 (47%)	0.075
Temperature (°C)				<0.01
<37.5 °C	74 (49.3%)	35 (70%)	40 (40%)	
37.5–38.0 °C	24 (16%)	8 (16%)	15 (15%)	
38.1–39.0 °C	41 (27.3%)	7 (14%)	34 (34%)	
>39.0 °C	11 (7.3%)	0	11 (11%)	
Systolic blood pressure <90 mmHg	2 (1.3%)	0	2 (1.33%)	0.085
Diastolic blood pressure <60 mmHg	14 (9.3%)	1 (2%)	13 (13%)	0.144
Peripheral oxygen saturation <93%	125 (83%)	39 (78%)	86 (86%)	<0.01
Heart rate >100 beats/minute	30 (20%)	6 (12%)	24 (24%)	0.022
Dyspnea	102 (68%)	71 (71%)	31 (62%)	0.281
Cough	97 (64.7%)	60 (60%)	37 (74%)	0.082
Sputum	52 (34.7%)	14 (28%)	38 (38%)	0.217
Chills	51 (34%)	11 (22%)	40 (40%)	0.021
Headache	52 (34.7%)	17 (34%)	35 (35%)	0.904
Fatigue	101 (67.3%)	27 (54%)	74 (74%)	0.019
Gastrointestinal symptoms	28 (18.7%)	9 (19%)	19 (19%)	0.883
Myalgia	55 (36.7%)	14 (28%)	41 (41%)	0.121
Rash	3 (2%)	0	3 (2%)	0.083
Duration from onset of symptoms to hospital admission (days)	4 (3–6)	4 (3–6)	4 (3–6)	0.859
Length of stay in hospital (days)	12 (8–16)	15 (13–19)	10 (6–14)	<0.001
Duration from ICU admission to death(days)	6.5 (3–8)	-	6.5 (3–8)	-

Data are expressed as absolute numbers and percentages (%), median (IQR) or mean (SD), as appropriate. Abbreviations: ICU—intensive care unit.

**Table 2:** Associated pathologies.

Pathology	Total (*n* = 150)	Survivors (*n* = 50)	Non-Survivors (*n* = 100)	*p* Value
Chronic obstructive pulmonary disease	24 (16%)	5 (10%)	19 (19%)	0.125
Diabetes	58 (38.7%)	18 (36%)	40 (40%)	0.683
Arterial hypertension	91 (60.7%)	22 (44%)	69 (69%)	0.004
Coronary heart disease	46 (30.7%)	10 (20%)	36 (36%)	0.045
Atrial fibrillation	25 (16.7%)	4 (8%)	21 (21%)	0.023
Cerebrovascular diseases	18 (12%)	5 (10%)	13 (13%)	0.597
Chronic heart failure	62 (41.3%)	15 (30%)	47 (47%)	0.042
Chronic liver diseases	12 (8%)	3 (6%)	9 (9%)	0.526
Chronic renal diseases	24 (16%)	1 (2%)	23 (23%)	<0.001
Malignancy	20 (13.3%)	7 (14%)	13 (13%)	0.866
Immunodeficiency	28 (18.7%)	10 (20%)	18 (18%)	0.769

**Table 3:** Laboratory findings.

Parameter	Total (*n* = 150)	Survivors (*n* = 50)	Non-Survivors (*n* = 100)	*p* Value
White blood cell count, ×10^9^/L				0.002
<4	12 (8%)	6 (12%)	6 (6%)	
4–10	87 (58%)	35 (70%)	52 (52%)	
>10	51 (34%)	9 (18%)	42 (42%)	
Neutrophil to lymphocyte ratio	8.3	7.7	8.4	0.022
Platelet count, ×10^9^/L	177	169	187	0.419
<150	102 (68%)	38 (76%)	64 (64%)	
Hemoglobin, g/dl	12	13	12	0.026
<12	71 (47.3%)	28 (56%)	43 (43%)	
C-reactive protein, mg/L	95.5	102	93	0.893
>5	144 (96%)	50 (100%)	94 (94%)	
D-dimer, mg/L	0.8	0.4	1.7	<0.001
>0.5	98 (63.5%)	21 (42%)	77 (77%)	
Interleukin-6, pg/mL	102	87	124	0.005
>1.8	150 (100%)	50 (100%)	100 (100%)	
Ferritin, ng/mL	568	471.5	682	<0.001
>350	126 (84%)	35 (70%)	85 (85%)	
Lactate dehydrogenase,	430	404.5	445	0.085
>430 U/L	75 (50%)	19 (38%)	56 (56%)	
Aspartate aminotransferase, U/L	43	42	43	0.203
>37	94 (62.7%)	32 (64%)	62 (62%)	
Alanine aminotransferase, U/L	38.5	41	38	0.193
>40	74 (49.3%)	27 (54%)	46 (46%)	
Total bilirubin, mg/dl	0.8	0.7	0.8	0.227
>1	49 (32.7%)	12 (24%)	37 (37%)	
Creatinine, mg/dl	0.9	0.9	1	0.011
>1.1	48 (32%)	11 (22%)	37 (37%)	
Urea, mg/dl	56	44	62.5	<0.01
>50	93 (62%)	18 (36%)	75 (75%)	
Blood sugar, mg/dL	137.5	137	140.5	0.973
>115	112 (74.7%)	40 (80%)	72 (72%)	
INR	1.1	1.1	1.1	0.011
>1.2	36 (24%)	4 (8%)	32 (32%)	
Imaging findings				
Ground-glass opacities	103 (68.7%)	30 (60%)	73 (73%)	0.121
Focal pulmonary infiltration	31 (20.7%)	16 (32%)	15 (15%)	0.028
Diffuse andbilateral pulmonary infiltration	50 (33.3%)	7 (14%)	43 (43%)	<0.001

**Table 4:** Therapeutic approach and complications.

	Total (*n* = 150)	Survivors (*n* = 50)	Non-Survivors (*n* = 100)	*p* Value
Treatments				
Mechanical ventilation				
Non-invasive	61 (40.7%)	9 (18%)	52 (52%)	<0.001
Invasive	105 (70%)	5 (10%)	100 (100%)	<0.001
Antiviral agents	136 (90.7%)	47 (94%)	89 (89%)	0.282
Antibiotics	139 (92.7%)	46 (92%)	93 (93%)	0.826
Glucocorticoids	127 (84.7)	46 (92%)	81 (81%)	0.049
Tocilizumab	125 (83.3%)	47 (94%)	78 (78%)	0.003
Complications				
Acute respiratory distress syndrome	116 (77.3%)	23 (46%)	93 (93%)	<0.001
Acute heart failure	12 (8%)	0	12 (8%)	<0.001
Acute kidney failure	29 (19.3%)	1 (2%)	28 (28%)	<0.001
Septic shock	7 (4.7%)	0	7 (7%)	0.008
Multiple organ dysfunction syndrome	39 (26%)	0	39 (26%)	<0.001

**Table 5:** Multivariable logistic regression analysis of mortality risk factors for patients with severe COVID-19 pneumonia.

Parameter	B	S.E.	Wald	*p*	Exp (B)	95.0% C.I. for EXP(B)	
						Lower	Upper
CRP	0.081	0.025	10.670	0.001	1.085	1.033	1.139
D-Dimer	2.262	0.732	9.546	0.002	9.603	2.287	40.325
Heart rate	0.230	0.072	10.091	0.001	1.259	1.092	1.451

**Table 6:** Correlations of biomarkers with predictors of poor prognosis.

	D-Dimer		C-Reactive Protein	
Parameter	r	*p*	r	*p*
Multiple organ dysfunction syndrome	0.198	0.015	0.199	0.015
Non-invasive mechanical ventilation	0.059	0.472	0.237	0.004
Invasive mechanical ventilation	0.366	<0.001	0.252	0.002
Use of glucocorticoids	−0.007	0.934	−0.054	0.513
Use of Tocilizumab	0.086	0.297	0.019	0.814
Admission to ICU	0.389	<0.001	0.335	<0.001
C-reactive protein	0.238	0.003	1	-
D-Dimer	1	-	0.238	0.003

**Table 7:** AUC detailed analysis: the biomarkers’ capacity in predicting admission to ICU.

Area Under the Curve					
Test Result Variable(s)	Area	Std. Error	Asymptotic Sig.	Asymptotic 95% Confidence Interval	
				Lower Bound	Upper Bound
C-reactive protein	0.707	0.042	<0.0001	0.624	0.790
D-Dimer	0.741	0.041	<0.0001	0.660	0.821

**Table 8:** Cut-off values for D-Dimer and C-reactive protein.

Criterion	D-Dimer Cut-Off (mg/L)	Se	Sp	C-Reactive Protein Cut-Off (mg/L)	Se	Sp
Se = Sp	0.74	65.7%	70.8%	48.5	61.8%	62.5%
Youden’s index (Maximum Se + Sp)	2.05	47.1%	92.7%	68.5	56.9%	85.7%
High-risk profile	0.41	80.4%	52.1%	23	80.1%	51.7%

Se = sensitivity; Sp = specificity.

## Data Availability

All necessary data is found within the text of the manuscript.
